# Illness Anxiety Disorder and Distress among Female Medical and Nursing Students

**DOI:** 10.2174/0117450179277976231115070100

**Published:** 2023-11-20

**Authors:** Sana Hawamdeh, Fatchima L. Moussa, Sami Al-Rawashdeh, Sajidah Al Hawamdih, Mahaman. L. Moussa

**Affiliations:** 1 Philadelphia, PA19104, USA; 2 Academy of Vocal Arts, Medical Surgical Department, College of Nursing, Princess Nourah bint Abdulrahman University, Riyadh, Saudi Arabia.; 3 Department of Community and Mental Health Nursing- Faculty of Nursing, The Hashemite University, P.O. Box 330127, Zarqa 13133, Jordan; 4 Applied Medical School- Luminus Technical University College (LTUC), Airport Road, Near Marj Al Hamam Bridge, Amman, Jordan; 5 College of Nursing, King Saud University, Riyadh, Saudi Arabia

**Keywords:** Illness anxiety disorder, Hypochondriasis, Medical students, Nursing students, Distress, Symptom disorder

## Abstract

**Objective:**

This study aims to compare the prevalence of illness anxiety disorder (IAD) and distress between medical and nursing students and examine their associations with students' characteristics.

**Methods:**

Cross-sectional data were collected using the Short Health Anxiety Inventory (SHAI, for measuring IAD) and the Medical Students' Disease (MSD) Perception and Distress Scales.

**Results:**

Two hundred and sixteen Medical students and 250 Nursing students were recruited from a public female university in Saudi Arabia. Their mean age was 21.27 years. The findings showed that the overall prevalence of IAD (SHAI scores ≥18) among the total sample was 38.8%, with a significantly lower prevalence in medical students compared to the prevalence in nursing students (57.2% *vs* 17.6%, respectively, *X*^2^**=**45.26, *p*<.001). Nursing students had significantly higher SHAI scores and lower MSD Perception scores than medical college students, but there were no significant differences among them in the MSD Distress scale. Significant differences in the main study variables scores were reported among nursing students but not among medical students, with the fourth-year level nursing students having higher SHAI and lower MSD Perception and perception scores than other nursing students.

**Conclusion:**

The highlights that medical and nursing students are susceptible to developing anxiety-related disorders and distress that may have negative impacts on their academic achievements and future careers. Both nursing and medical faculty should help in identifying strategies to support the students' mental health and well-being.

## 1 INTRODUCTION

Illness anxiety disorder (IAD) is a somatic symptom disorder, previously known as hypochondriasis [1], characterized by a high level of anxiety and fear and the belief of having or acquiring a serious illness [2]. The affected individual suffers a high level of health anxiety, performs excessive health-related behaviors (*e.g*., repeatedly checks their body for signs of a disease or illness), and is easily alarmed about their health status [1, 3]. IAD is also known as a Medical student's disease and health-related anxiety [4], as it's prevalent among them [5]. The IAD prevalence among medical students has been reported in countries such as Malaysia, Australia, and India. In general, practice students were 37.2%, 18.6%, and 3.2%, respectively [6-8]. In Saudi Arabia, a cross-sectional study showed that the overall prevalence of IAD among 400 medical students was 3.4% [9]. Examining IAD in medical students or health professions students, in general, is important as they could develop IAD attributed to the diseases that they are studying [10] or incomplete medical knowledge acquired during their gradual learning process in theoretical or clinical courses [5] as well as exposure to high numbers of diseases and health conditions.

Stress experienced in medical schools and students' other factors, such as personality and previous mental health problems, may negatively impact their mental health [11]. A recent systematic review of the prevalence rate of anxiety among medical and non-medical students found that Middle Eastern and Asian medical students had the highest prevalence of anxiety [12]. As for nursing students, the focus was on the source of stressors and coping mechanisms and the association of stress and anxiety with clinical practice [13]. Scarsella and colleagues (2016) showed that most individuals suffering from IAD also have to underline psychiatric illnesses [14].

These health concerns and sources of stressors may interfere with students' academic performance [15]. Early prediction of IAD can help minimize the risk factors [16]. It was revealed in a study by Sharma and Wavare (2013) that medical students who are depressed or lack support from their environment and who communicate less with others tend to feel isolated and withdrawn from others [17]. Thus, healthcare providers, including physicians and nurses in the universities clinics and the instructors, need to be more aware of the student's struggles to assist them accordingly and avoid any problems in the future [18]. It has been recommended for nurses to have their health anxiety symptoms under control to avoid feeling overwhelmed, which, if not controlled, can lead to resignation and then shortage [19]. This could also be applied to the students, either medical or nursing students, as a high level of IAD, could negatively influence the retention of students in their study as well as their willingness to continue working in the healthcare field. IAD may affect a person's health, self-medication and [20] interpersonal relationships [21]. IAD has been shown to be associated with low life satisfaction and high alexithymia levels in junior nursing students [22]. IAD also may negatively affect the safety of patients and the safety culture in clinical areas and in educational institutions.

There is a clear need to monitor and implement measures to support students exhibiting health anxiety. The students play a significant role and are essentially the future of the healthcare industry, so they should be valued and assisted in case of need [17]. Different results have been yielded from many studies about IAD prevalence among medical students. In addition, very few studies addressed the prevalence of IAD among nursing students [5, 22]. There are several factors to be considered in health anxiety, such as personality, mood characteristics and ability to withstand emotional and physical distress [23]. Identifying the prevalence of IAD symptoms in different vulnerable populations could be a great strategic way to develop programs and interventions that may reduce the prevalence and prevent it in the future. It is also important to compare both medical and nursing students to understand the prevalence of IAD among those two groups as previous studies either examine IAD in general students or in a specific specialty area. In normal circumstances, medical and nursing students may have different sources of anxiety, such as the complicated structure of courses, fear of failing exams, academic competitions, and clinical training. Thus, this study aims to determine the prevalence of IAD and distress among medical and nursing students in a female public university in Saudi Arabia.

## 2 MATERIALS AND METHODS

### 2.1 Design

An analytical descriptive comparative cross-sectional design was employed to complete this study.

### 2.2 Participants and Study Procedure

All students from the first to the fourth year from the nursing college and medical college (from a public university for women in Saudi Arabia) were invited to participate in this study. The university is located in Riyadh, the capital of Saudi Arabia and offers diploma to postgraduate degrees from different humanities, sciences, and health sciences colleges. All Bachelor's degree students (900 students) in the mentioned colleges are considered eligible to participate. The required sample size was calculated using the ANOVA test (used to examine the difference in IAD symptoms, MSD Perception and Distress among each medical and nursing student based on their years of study (4 groups)) with a medium effect size (0.25), p-value 0.05, and a power of 0.90. The required sample size from each nursing and medical student was 232 students. After explaining the aim of the study and obtaining their written informed consent to participate, students were informed to fill out the questionnaire during their free time. Of the invited students, 250 nursing students and 216 medical students completed a self-administered questionnaire. Information regarding their rights (*e.g*., confidentiality, termination participation, and voluntary participation) was thoroughly explained to the participants.

### 2.3 Instruments for Data Collection

The self-administered questionnaire consisted of a demographic data section and two scales the Short Health Anxiety Inventory (SHAI) and the Medical Students' Disease (MSD) Perception and Distress Scale. Students also answered additional questions about whether they visited a doctor within the past six months and whether they were addicted to substances, such as cigarette smoking or drugs or not.

The SHAI is a short version of the original, full-length Health Anxiety Inventory and is composed of 18 items [24]. It is used to measure the IAD level. Items 1 to 14 measure the presence of health anxiety, and Items 15 to 18 measure only the fear of morbidity associated with severe illness. Its internal reliability (Cronbach's alpha) ranged from 0.87 to 0.95 among the student population [25, 26]. The responses of the items ranged from 0 “not at all or rarely” to 3 “most of the time” [27]. The scores of the 18 items are summed to yield a total score that could range between 0 and 54. A score of 18 or above was considered a high level of health-related anxiety [7, 28].

In addition to the SHAI, the Medical Students Disease (MSD) scale was used in this study as it measures the entities or components of the IAD or health-related anxiety; MSD perception or cognitive component (the first five items) and MSD distress component (the last five items) [24]. The perception or cognitive component is related to students' thoughts of having the disease/s studied, while the distress component is associated with the anxiety developed as a result of the cognitive component [29]. The item's responses ranged from 1 (definitely false) to 5 (definitely true), with total scores calculated for both cognitive and distress components separately by summing the responses of the pertinent items. Each subscale score could range between 1 and 25, with higher scores indicating worse cognitive or distress components. The MSD scale demonstrated excellent internal reliability (Cronbach's alpha), ranging from 0.78 to 0.90 across student samples [4, 29]. The questionnaire was also pilot-tested to ensure its reliability, validity, appropriateness, and relevance, with reported alpha values of (0.84) and (0.72) for cognitive and distress components, respectively.

### 2.4 Data Analysis

Data were analyzed using SPSS v23.0 (SPSS Inc., Chicago, IL, USA). All continuous data were presented as mean ± SD, while categorical data were presented as frequencies and percentages. Independent sample t-tests were used to examine the difference scores between the two groups, including comparing IAD symptoms, MSD Perception and Distress between medical and nursing students. The Chi-square test was used to examine the percentages of high and low SHAI scorers between medical and nursing students. The one-way ANOVA was used to examine the difference in IAD symptoms, MSD Perception and Distress among students based on their years of study (more than 3 groups) with the Scheffe test as a post hoc test. Further, each student's group scores were compared based on the student's responses to the extra questions using the Independent t-tests. P-value was set at 5% for all analyses for significance level.

### 2.5 Ethical Consideration

The study was conducted according to the principles of the Helsinki Declaration and was approved by the Institutional Review Board (IRB) of a public university in Saudi Arabia.

### 3 RESULTS

Four hundred sixty-six female students participated in the study (216 from the Medical College and 250 from the Nursing College). The mean age of the participants was 21.27 years (range 19-27 years). The students who participated in the study were stratified by their academic level (years of study, Table **1**). As shown, none of the first-year nursing students participated in the study.

Table **1** also shows the comparisons of the main study variables of medical and nursing students based on their academic level (academic year). For the medical college students, there were no significant differences in students' scores on all of the examined variables based on the students' academic level using the one-way ANOVA test (all p-values > 0.05). Among nursing students, the one-way ANOVA test results indicated significant differences in the three main variables' scores based on the students' academic level (all p-values < 0.01). The post hoc test showed that the SHIA means scores were significantly higher among nursing students in the fourth year (22.13±4.20) compared to the second (15.92 ±6.40) and third year (14.90±6.76). Post hoc tests also indicated that the 4th-year level students had significantly lower scores on MSD perception than the students in the 2nd- and 3rd-year level students. Also, the 4th-year level students had significantly lower scores on MSD distress than the 2nd-year students.

The scores of students on the main study variables in both colleges were compared using the independent sample t-test. Nursing students scored on average 5.7 scores higher on SHAI than the medical college students (18.2±6.6 *vs* 12.5±6.3, t (464) = -9.49, p<0.01). However, medical college students significantly scored higher than nursing students on MSD Perception scale scores (14.80±4.08 *vs* 12.55±4.44, t (464) = 5.66, p-value <0.01). There were no significant differences in MSD Distress scores in medical *vs* nursing students (13.58±4.30 *vs* 12.90±4.17, t (464) = 1.73, p-value=0.84).

The overall prevalence of IAD (SHAI scores ≥18) among the total sample was 38.8% (Table **2**). The high IAD scorers (SHAI scores ≥18) were more common among nursing students than among medical college students (57.2% *vs* 17.6%, respectively, *X*^2^**=**45.26, p-value <.001).

Among the 250 nursing students, 139 (55.6%) reported visiting doctors during the last 6 months, while 144 (66.7%) of medical college students did. In each college, those who visited a doctor during the last six months were compared with those who did not use the independent sample t-test. There were no significant differences in students' scores on the main study variables between those who visited a doctor and those who did not in both nursing and medical college students (Table **3**). Similar results were also reported related to the comparisons between students who were addicted to substances (such as Cigarette drugs, *etc*) and those who did not (Table **3**).

## 4 DISCUSSION

This study identified the prevalence of IAD in medical and nursing student samples in Saudi Arabia. Health anxiety among medical students was identified in previous studies done in Croatia and Malaysia [7, 28, 30, 31]. The results are similar to previous local studies that show the prevalence of IAD (hypochondriasis) among medical students [28, 30]. This indicates that medical students are more likely to experience health anxiety and IAD symptoms. Interestingly, the study found a higher prevalence of IAD among nursing students. Studies showed that medical students, nursing students, public health students, and pharmacology students are more prone to developmental distress due to the competition they may face, the work required, and the anxiety associated with new clinical experiences, which creates academic and social stress [32, 33]. Notably, transient IAD symptoms are common in health sciences students [29]. While these students start learning about diseases yet have an incomplete understanding of them, they may compare their bodily symptoms or even imagined signs, paying selective attention to similarities and overlooking inconsistencies. Azuri *et al*. (2010) study reported that medical students with IAD symptoms, especially first-year students, are more likely to seek professional consultation, and they visit specialists 1.6 times more than those without symptoms [34].

Moss-Morris and Petrie (2001) investigated the presence of IADs by looking at its two components: cognitive and distress. They showed that the distress component decreased over time [29]. The present study revealed a rise in the mean scores of the mental (IAD- Perception) in medical students than in nursing students. Nevertheless, the differences in scores across the different cohorts of nursing students were statistically significant for the IAD Perception and Distress components throughout the years. The present study found that the 2nd-year nursing students scored higher on perception and distress components than 1st-year nursing students relatively because the second-year students may demonstrate excellent knowledge and a good understanding of basic medical sciences and the disease process. However, these results are different in the third and fourth years; distress and perception components for medical and nursing students were less noticeable. This indicates that IAD symptoms are more likely to be alleviated by detailed medical knowledge gained during later years, which may prevent students from getting greatly distressed and anxious by the thoughts of having contracted the disease being studied [29, 33].

This study also highlights the percentage of students who visited doctors at least once in the past six months. It was observed in this study that more medical students had seen or consulted their doctors than nursing students. This is more than the reported percentage among Saudi medical students (50%) [28] and less than the percentage (73%) stated by Zahid *et al*. (2016) study among Pakistani medical students [4]. There is a positive correlation between health anxiety and increased student visits to medical doctors during the past six months. However, the study showed that medical students scored less than nursing students concerning health anxiety after visits to medical doctors. This result is similar to the findings of Moss-Morris and Petrie (2001), in which the IAD perception and distress results were not significantly associated with the number of visits to doctors for both groups of students. Several studies have investigated students' coping mechanisms for managing IAD symptoms, including consultation with professionals or non-professionals [31, 35].

This study has shown that a small fraction of medical and nursing students have addictions. Although only a small fraction of students admitted to having addictions, this fraction had a significantly higher mean score on the SHAI and the IAD Distress Scale. Smoking in the current study was significantly associated with health anxiety, and this finding was inconsistent with Ezmeirlly and Farahat's (2019) study on Saudi medical students. It is significant to conduct sessions to teach the students some coping strategies and better understand anxiety. In addition to increasing the students' repertoire of coping mechanisms, researchers should conduct more studies related to IAD symptoms, comparing nursing with medical students in Arab countries and applying similar studies to large populations. They should include female and male students to investigate the gender effect.

The study presents some limitations. First, the cross-sectional design of the study precludes causality. Second, IAD is measured by the subjective report and only provides a glance at the symptoms. Further research is needed to better measure IAD at the national level to understand the burden among medical and nursing students. Nevertheless, this study provides additional insights into the prevalence of IAD in medical and nursing student samples in Saudi Arabia.

This study has several implications. School nurses, educators, and researchers have to pay attention to this group by developing and testing programs that support students, especially in the early years of their studies—implementation of counseling programs and providing educational sessions related to developing skills to deal with IAD symptoms. Attention should also be focused on nursing students since they have a significant proportion of students who have IAD symptoms.

## CONCLUSION

In conclusion, the study showed that nursing students in Saudi Arabia have high IAD symptoms compared to medical students. The findings are unexpected but show that nursing students are also susceptible to developing anxiety-related issues or IAD symptoms. Both nursing and medical faculty should be aware of the negative impact of stress on students, academic achievements, and future careers. Teachers' role is crucial in identifying strategies to support the students' mental health and well-being.

## AUTHORS' CONTRIBUTION

• **Conceptualization**: Sana Hawamdeh;

• **Methodology**: Sana Hawamdeh & Fatchima Moussa;

• **Formal analysis**: Sana Hawamdeh, Fatchima Moussa, & Sami Al-Rawashdeh;

• **Investigation**: Sana Hawamdeh, Fatchima Moussa, & Sajidah Al- Hawamdih;

• **Resources**: Sajidah Al- Hawamdih & Mahaman Moussa;

• **Data Curation**: Fatchima Moussa & Mahaman Moussa;

• **Writing**:

a) Original draft preparation: Sana Hawamdeh, Sajidah Al- Hawamdih & Fatchima Moussa;

b) Review and editing: Fatchima Moussa, Mahaman Moussa, & Sami Al-Rawashdeh;

c) Visualization: Sana Hawamdeh & Fatchima Moussa;

d) Supervision: Sana Hawamdeh, Fatchima Moussa, & Sami Al-Rawashdeh;

All authors have read and agreed to the published version of the manuscript.

## Figures and Tables

**Table 1:** The differences among medical and nursing students in terms of the main study variables based on their level of study.

**Medical College**								
-	**All**	**1st Year**	**2nd Year**	**3rd Year**	**4th Year**	F-test Value*	** p-value **	**Post Hoc** **(Significant Differences)**
**Scale/ n**	**216**	**50**	**53**	**63**	**50**	-	-	-
**SHAI, Mean (SD)**	12.50 (6.26)	12.86 (6.24)	13.04 (6.45)	13.03 (6.71)	10.90 (5.37)	1.43	0.23	-
**MSD Perception Scale, Mean (SD)**	14.80 (4.08)	15.14 (4.40)	14.81 (4.17)	14.79 (4.00)	14.45 (3.80)	0.231	0.875	-
**MSD Distress Scale, Mean (SD)**	13.58 (4.30)	14.36 (4.33)	13.98 (4.74)	13.71 (4.03)	12.22 (3.90)	2.44	.07	-
	-	-	-	-	-	-	-	-
**Nursing College**								
-	**All**	**1st Year**	**2nd Year**	**3rd Year**	**4th Year**	F-test Value*	** p-value **	**Post Hoc**
**Scale/ n**	**250**	**0**	**77**	**70**	**103**	-	-	-
**SHAI, Mean (SD)**	18.20 (6.59)	-	15.92 (6.40)	14.90 (6.76)	22.13 (4.20)	42	<0.01	4^th^ year> 3^rd^ year 4^th^ year> 2^nd^ year
**MSD Perception Scale, Mean (SD)**	12.55 (4.44)	-	14.32 (4.49)	15.19 (4.22)	9.44 (2.00)	67.22	<0.01	4^th^ year< 3^rd^ year 4^th^ year< 2^nd^ year
**MSD Distress Scale, Mean (SD)**	12.90 (4.17)	-	15.04 (4.70)	13.90 (4.73)	10.61 (1.90)	35.31	<0.01	4^th^ year< 2^nd^ year

**Abbreviations: **SHAI=Short Health Anxiety Inventory; MSD= Medical Students' Disease; *One-way ANOVA test.

**Table 2:** SHAI scores categories for medical and nursing students.

**SHAI Score Categories**	-	**Colleges**		**X^2^ test** **value**	** p-value **
	**Total**	**Medical (216)**	**Nursing (250)**		
	**N(%)**	**N(%)**	**N(%)**		
**SHAI <18 (low)**	285 (61.15)	178 (82.4)	107 (42.8)	45.26	<.001
**SHAI ≥18 (high)**	181 (38.8)	38 (17.6)	143 (57.2)	-	-

**Abbreviations: **SHAI=Short Health Anxiety Inventory.

**Table 3:** differences among medical and nursing students in terms of the main study variables based on their visiting a doctor during the last 6 months and addictive behavior.

-	**Nursing Students (n=250)**			**Medical Student (n=216)**		
**Scales scores,** **Mean (SD)**	**Visited a doctor in the previous 6 months**					
	**Yes** **139 (55.6%)**	**No** **111 (44.4%)**	**P-value**	**Yes** **144 (66.7%)**	**No** **72 (33.3%)**	**P-value**
**SHAI**	18.41 (6.56)	17.91 (6.64)	0.55	12.69 (5.99)	12.11 (6.79)	0.52
**MSD Perception scale**	12.84 (4.34)	12.19 (4.54)	0.25	14.74 (4.13)	14.89 (3.92)	0.80
**MSD Distress scale**	12.64 (4.55)	13.22 (3.63)	0.25	13.80 (4.30)	13.15 (4.30)	0.29
-	**Addiction (Cigarette smoking, drugs, etc.)**					
-	**Nursing Students (n=250)**			**Medical Student (n=216)**		
-	**Yes** **9 (3.6%)**	**No** **241 (96.4%)**	**P-value**	**Yes** **7 (3.2%)**	**No** **209 (96.8%)**	**P-value**
**SHAI**	18.19 (10.86)	18.19 (6.41)	0.99	15.57 (4.86)	12.40 (6.29)	0.19
**MSD Perception**	14.33 (6.36)	12.48 (4.35)	0.22	13.71 (5.82)	14.82 (3.99)	0.48
**MSD Distress**	13.89 (6.07)	12.85 (4.09)	0.47	14.00 (4.47)	13.56 (4.30)	0.79

**Abbreviations: **SHAI=Short Health Anxiety Inventory, MSD= Medical Students' Disease.

## Data Availability

The data presented in this study are available on request from the corresponding author [S.A-R]. The data are not publicly available due to privacy and ethical concerns.
